# Vital capacity and valvular dysfunction could serve as non-invasive predictors to screen for exercise pulmonary hypertension in the elderly based on a new diagnostic score

**DOI:** 10.34172/jcvtr.2021.05

**Published:** 2021-01-30

**Authors:** Simon Wernhart, Jürgen Hedderich, Eberhard Weihe

**Affiliations:** ^1^Department of Cardiology, Fachkrankenhaus Kloster Grafschaft, Schmallenberg, Germany; ^2^University Hospital Essen, University Duisburg-Essen, West German Heart- and Vascular Center, Department of Cardiology and Vascular Medicine, Hufelandstrasse 55, 45147 Essen, Germany; ^3^Medistat-Biomedical Statistics, Medistat GmbH, Kronshagen, Germany; ^4^Institute of Anatomy and Cell Biology of the Philipps-University Marburg, Germany

**Keywords:** Exercise Pulmonary Hypertension, Elderly, Valvular Dysfunction, Vital Capacity

## Abstract

***Introduction:*** Exercise pulmonary hypertension (exPH) has been defined as total pulmonary resistance (TPR) >3 mm Hg/L/min and mean pulmonary artery pressure (mPAP) >30 mm Hg, albeit with a considerable risk of false positives in elderly patients with lower cardiac output during exercise.

***Methods:*** We retrospectively analysed patients with unclear dyspnea receiving right heart catheterisation at rest and exercise (n=244) between January 2015 and January 2020. Lung function testing, blood gas analysis, and echocardiography were performed. We elaborated a combinatorial score to advance the current definition of exPH in an elderly population (mean age 67.0 years±11.9). A stepwise regression model was calculated to non-invasively predict exPH.

***Results:*** Analysis of variables across the achieved peak power allowed the creation of a model for defining exPH, where three out of four criteria needed to be fulfilled: Peak power ≤100 Watt, pulmonary capillary wedge pressure ≥18 mm Hg, pulmonary vascular resistance >3 Wood Units, and mPAP ≥35 mm Hg. The new scoring model resulted in a lower number of exPH diagnoses than the current suggestion (63.1% vs. 78.3%). We present a combinatorial model with vital capacity (VC_max_) and valvular dysfunction to predict exPH (sensitivity 93.2%; specificity 44.2%, area under the curve 0.73) based on our suggested criteria. The odds of the presence of exPH were 2.1 for a 1 l loss in VC_max_ and 3.6 for having valvular dysfunction.

***Conclusion:*** We advance a revised definition of exPH in elderly patients in order to overcome current limitations. We establish a new non-invasive approach to predict exPH by assessing VC_max_ and valvular dysfunction for early risk stratification in elderly patients.

## Introduction

Pulmonary hypertension (PH) and exercise pulmonary hypertension (exPH) are predictors of morbidity and mortality.^1-6^ Borderline PH at rest seems to expose patients to similar limitations during exercise as ‘real’ PH does, and should prompt immediate exercise testing.^7^ Pre-emptive measures are required to detect this condition, raising the question of whether early treatment could provide clinical benefit as recently shown in a small cohort of patients.^8-11^ However, before standardised treatment can be initiated, a precise and reliable definition of exPH is needed, since it seems to be a unique pathological entity, with patients displaying different alterations in biochemical pathways than resting PH patients and healthy controls.^12,13^ Additionally, the kinetics of hemodynamic recovery seem to vary between different types of PH and exPH.^14^

Several factors appear to limit the cardiovascular response to increased right ventricular (RV) afterload during exertion. A low diffusion capacity of carbon monoxide (DLCO) was suggested to identify exPH patients with parenchymatous lung disease,^15^ while failure of RV contractility increase during exercise may lead to RV-PA (pulmonary artery) uncoupling and exPH.^16^

Until 2009 exPH was defined as an invasively measured mPAP>30 mm Hg.^17,18^ This definition, however, was abandoned due to its dependence on age and exercise capacity, with a high number of false positive results.^19,20^ A French group established a new definition for exPH using a combination of mPAP>30 mm Hg and total pulmonary resistance (TPR, defined as mPAP/CO, cardiac output).^19^ TPR represents the steepness of PAP-increase during exercise. A TPR>3 mm Hg/L/min and an mPAP>30 mm Hg during exercise may currently be the best way to define exPH.^21^ These cut-off values have been shown to be independently associated with cardiovascular event-free survival (hazard ratio 2.03) in a population of patients with preserved ejection fraction during a mean follow-up of 3.7 years,^22^ which confirms the clinical relevance of further investigating patients with unclear dyspnea and ambiguous resting right heart catheterisation (RHC). This definition can be seen as the current gold standard to define exPH. Concerns have been raised about its applicability in elderly (>60 years), multi-morbid, and frail patients with lower cardiac output and muscular limitations during exercise.^23^ We aimed to fill this gap by investigating elderly patients reporting to our medical unit for unclear dyspnea who received RHC at rest (rRHC) and exercise (exRHC).

A criterion of exercise capacity in cardiopulmonary exercise testing (CPET) is maximal power of performance [W]. This, however, has not found its way into exPH definitions so far. Thus, we aimed to develop a new combinatorial definition of exPH taking into account hemodynamic parameters of RHC as well as maximal power of performance. Our diagnostic score should be compared to the current standard (TPR>3 mm Hg/L/min and an mPAP>30 mm Hg). We hypothesise that our new score will reduce the number of exPH diagnoses in the elderly population compared to the currently suggested definition. Finally, we analysed non-invasive parameters in terms of their potential to predict exPH based on this new model and aiming to avoid invasive RHC testing in elderly patients without missing the correct diagnosis (high sensitivity is necessary).

## Materials and Methods

### Study design and participants

We performed a retrospective, exploratory study screening a total of 244 patients who had reported to our clinic between January 2015 and January 2020 for diagnostics of unclear dyspnea and who had received rRHC and exRHC following inconclusive non-invasive pneumological and cardiological investigation (dyspnea out-of-proportion of the underlying disorder). The medical decision for the necessity of invasive RHC evaluation was made by an experienced cardiologist after informed consent was signed by the patient or legal guardian. Patients had to be at least 50 years of age; clinically stable medical comorbidities were no exclusion criteria for participation as we wanted to access real-life data to elaborate a definition for exPH. As exPH can occur in the presence or absence of resting PH, patients were included regardless of their resting mPAP (and thus the presence of resting PH). Patients who did not fulfil the inclusion criteria and/or were unable or unwilling to provide informed consent for RHC investigation were excluded from the study.

**Figure 1:**
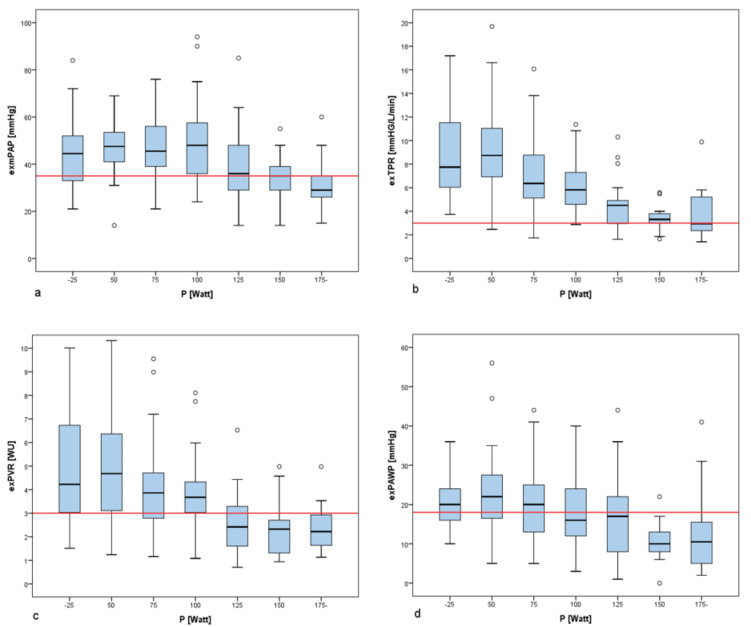
Hemodynamic variables across various peak performance strata

From 244 patients undergoing exRHC the development of pulmonary capillary wedge pressure (exPAWP), mean pulmonary artery pressure (exmPAP), pulmonary vascular resistance (exPVR), and the maximal performance (in Watts, W) were analysed during exercise (Figure 1). For each criterion one point was awarded; exPH was diagnosed if three or four points crossed the defined thresholds. In case of exPVR, exPAWP and exmPAP, we aimed to set the thresholds of our combinatorial score either at the observed deflection points across the achieved workload or, in the absence of a visible deflection point, at established thresholds in the literature.^17^ As we wanted to include the parameter maximal performance as a single factor, a maximal workload of 100W or less was deemed as a relevant limitation of cardiorespiratory fitness and was awarded one point. Furthermore, we chose 100W analogous to the suggested workload to define and assess non-invasive exercise hypertension (or exaggerated blood pressure response) during bicycle ergometry.^24^ ExRHC was performed on a bicycle ergometer directy after the resting measurements, either in patients without (<25 mm Hg) or moderately elevated mPAP (25-35 mm Hg) at rest. PH at rest was defined according to the suggestions of the Task Force of the 6th World Symposium on Pulmonary Hypertension, during which the mPAP threshold was lowered to 20 mm Hg.^25^ We performed exPH in the presence of moderate PH at rest to analyse the slope of mPAP increase during low-dose exercise (25-50W) in order to assess the effect of pulmonary vasodilatation at an intensity which represents everyday activities. There was no predetermined workload to achieve and patients in both groups were asked to exercise until maximal exertion, or the occurrence of medical reasons to prematurely terminate the stress test (such as ventricular tachycardia, a drop of systolic blood pressure >20 mm Hg , angina pectoris, dizziness, electrocardiographic signs of ischemia). In both groups, starting at a workload of 25W, an increase of 25W every two minutes was performed until maximal exertion.

Progression of coronary artery disease and acute infection had to be excluded invasively or non-invasively prior to RHC. Acute heart failure had to be clinically excluded. In case of fluid retention pharmacological (mainly using intravenous diuretics) or invasive (puncture of pleural effusions) measures had to be taken to ensure recompensation prior to RHC. We only included patients who had received lung function testing, transthoracic echocardiography, and arterial blood gas analysis within 24 hours of RHC to avoid a bias created by a different fluid status (both vital capacity, VC_max_, and echocardiographic systolic pulmonary artery pressure, sPAP, are influenced by fluid status).

Pulmonary disease was defined as relevant restrictive (total lung capacity, TLC, < Lower Limit of Normal, LLN)^26^ or obstructive (ratio between forced expiratory pressure in one second, FEV_1_/vital capacity during forced expiration, FVC. The ratio had to be <LLN)^26^ ventilation deficits or emphysematous aspects (diffusion capacity of carbon monoxide, DLCO <80%) at lung function testing (LFT), while cardiac disease was defined as the presence of coronary artery disease, cardiomyopathy, relevant valvular heart disease (≥ grade II stenosis or insufficiency of transthoracic echocardiography), or chronic heart failure (≥ NYHA II).

This study was conducted in accordance with the amended Declaration of Helsinki, and ethical approval was obtained (Ethics Committee University Munster, Germany, 2020-417-f-S). Written RHC consent had to be given separately.

### Assessment

Oxygen content (c_a_O_2_) of resting blood gas analysis was calculated according to the formula: SaO_2_ (%) x Hb (g/dL) x 1.34 (mL/g) + p_a_O_2_ (mm Hg) x 0.0031 (1/ mm Hg *mL/dL). Capillary partial pressure of oxygen (p_a_O_2_) and carbon dioxide (p_a_CO_2_) were also measured. LFT was done following established guidelines.^26^ Transthoracic echocardiography was performed by experienced cardiologists. Left ventricular ejection fraction (LVEF) was determined using the eyeballing method and separated into >50%, 40-50% and <40%. Right ventricular function (RVEF) was assessed by eyeballing and measuring tricuspid annular plane systolic excursion (TAPSE) ^27^ and systolic pulmonary artery pressure (sPAP) was determined by estimating RA pressure and calculating the pressure gradient from tricuspid regurgitation velocity according to established guidelines.^28^ Categorisation of valve dysfunction was done according to current guidelines.^29,30^

RHC was performed in a supine position with a fluid-filled 7 French Swan-Ganz catheter. Right brachial or internal jugular veins were used for access under sonographic guidance. Systemic blood pressure was taken using a cuff sphygmomanometer. Midchest position was used as zero reference. Right atrial (RA), right ventricular (RV), pulmonary artery (PA) and wedge (pulmonary capillary wedge pressure, PAWP) pressures were registered consecutively at the end of expiration during stable heart rate and mPAP-values. Appropriate wedge position had to be confirmed by deflating the balloon and immediate demonstration of a PA-curve profile. Mixed venous blood was taken and systemic vascular resistance (SVR) and pulmonary vascular resistance (PVR) were calculated (PVR=mPAP-PAWP/CO) and SVR=MAP-CVP/CO; CVP being central venous pressure and CO being cardiac output. We used an exercise protocol starting with a 2min warm-up phase at 0W followed by a 25W increment every 2min until exhaustion or contraindication for continuation (sudden drop in systolic blood pressure>20 mm Hg, sustained ventricular tachycardia, acute chest pain, new onset left bundle branch block) occurred. Patients were instructed to cycle at 60 revolutions/min. Cardiac output was measured with thermodilution in case of minor valve dysfunction ^31^ and by (additionally) applying Fick’s law (CO= VO_2_/avDO_2;_ oxygen consumption/ arterio-venous difference in oxygen) in advanced tricuspid valve insufficiencies (54.1% of patients with severe valve dysfunction), since thermodilution may produce lower results in relevant (at least grade two) tricuspid regurgitation.^32^ At least two measurements within 10% range had to be documented.

### Statistical analysis

Descriptive and exploratory analyses of data were performed with SPSS (IBM Corp. Released 2016. IBM SPSS Statistics for Windows, Version 24.0. Armonk, NY: IBM Corp.) The Kolmogorov-Smirnov test (including Lilliefors significance correction) and Shapiro-Wilk test were used for normality testing. Differences between subgroups were analysed using the Mann-Whitney U test and chi-squared test (with Yates’ continuity correction where appropriate) to investigate explanatory variables. Analysis of associations was performed using Spearman’s Rho correlation coefficients and multivariable logistic regression modelling was done with functions from the R-Program.^33,34^ Missing data were replaced by regression imputation. Alpha was set at .05. We opted to use an in/exclusion algorithm (using the Akaike information criterion (AIC)) to elucidate the most relevant variables for the prediction model.

Area under curve (AUC) was measured to discriminate the capability of the final model from a receiver operating characteristics (ROC) analysis. In order to obtain an estimate of sensitivity and specificity from logistic regression modelling the cut-off value was set as 0.5, and searching for an optimal cut-off in VC_max_ alone was done with minimal ROC distance. Odds Ratios (OR) were transformed from the regression coefficients and estimated probabilities of exPH were illustrated. Predictive capabilities of the model were visualised in a nomogram. Cut-offs to determine exPH were chosen based on clinical relevance and visual inspection of diagrams. The study variables were analysed and cut-off values were set at the respective deflection points across the achieved peak performance.

## Results

A total of 244 patients were initially evaluated. 36.9% of the entire population (mean age 67.0 years±11.9) had PH at rest, 12.3% had precapillary, 12.7% postcapillary and 11.9% combined pre- and postcapillary PH according to current guidelines.^17^ Baseline data of rRHC and exRHC are shown in Table 1.

**Table 1:** Baseline data of resting (rRHC) and exercise right heart catheterisation (exRHC, n=244)

		**n**	**Mean**	**SD**	**Median**	**IQR**
mPAP, mm Hg	rRHC	244	24.3	10.2	22.0	12.0
	exRHC	244	44.6	14.2	44.0	18.2
Pulse Pressure, mm Hg	rRHC	244	20.8	10.0	19.0	10.3
	exRHC	244	39.3	15.3	37.0	21.0
PAWP, mm Hg	rRHC	244	12.8	6.3	12.0	9.0
	exRHC	238	19.0	9.2	19.0	12.0
PVR, WU	rRHC	242	2.6	2.2	2.1	1.8
	exRHC	232	4.0	2.0	2.1	1.8
CO, L/min	rRHC	244	4.8	1.3	4.7	1.6
	exRHC	236	7.3	2.6	7.0	3.6
TPR, mm Hg/L/min	rRHC	244	5.6	3.3	4.8	3.1
	exRHC	236	7.2	4.1	6.2	4.3

Abbreviations: mPAP,mean pulmonary artery pressure; rRHC,resting right heart catheterisation; exRHC, exercise right heart catheterisation; PAWP, pulmonary capillary wedge pressure ;PVR, pulmonary vascular resistance at rest; CO, cardiac output; TPR, total pulmonary resistance at rest; SD,standard deviations; IQR, interquartile range

### Step 1. Comparison of variables from exRHC with the current ‘gold standard’

In order to define an alternative score for exPH we analysed the prevalence of currently used cut-off values during exercise (exPAWP, exmPAP, exTPR, exPVR) and the currently suggested ‘gold standard’ exTPR>3 mm Hg/L/min and exmPAP>30 mm Hg ^19^ in our population (Table 2).

**Table 2:** Descriptive data of hemodynamic variables (n=244)

exPVR	<3WU: n=84 (36.1%)	>3WU: n=149 (63.9%)
exPAWP	<20 mm Hg : n=133 (55.9%)	>20 mm Hg : n=105 (44.1%)
exTPR	<3 mm Hg/L/min: n=22 (9.3%)	>3 mm Hg/L/min: n=214 (90.7%)
exmPAP	<30 mm Hg : n=42 (17.2%)	>30 mm Hg : n=202 (82.8%)
Combined model: exTPR + exmPAP	< 3 mm Hg/L/min+ <30 mm Hg : n=53 (21.7%)	>3 mm Hg/L/min + >30 mm Hg : n=191 (78.3%)

Abbreviations:exPVR, pulmonary vascular resistance at peak exercise; exPAWP, pulmonary capillary wedge pressure at peak exercise; exmPAP, mean pulmonary artery pressure at peak exercise

### Step 2. Formation of a combinatorial diagnostic score for exPH

We analysed the hemodynamic variables exmPAP, exTPR, exPVR, and exPAWP and their relationships to the achieved power [W] (see Figures 1a-d).

**Figure 2:**
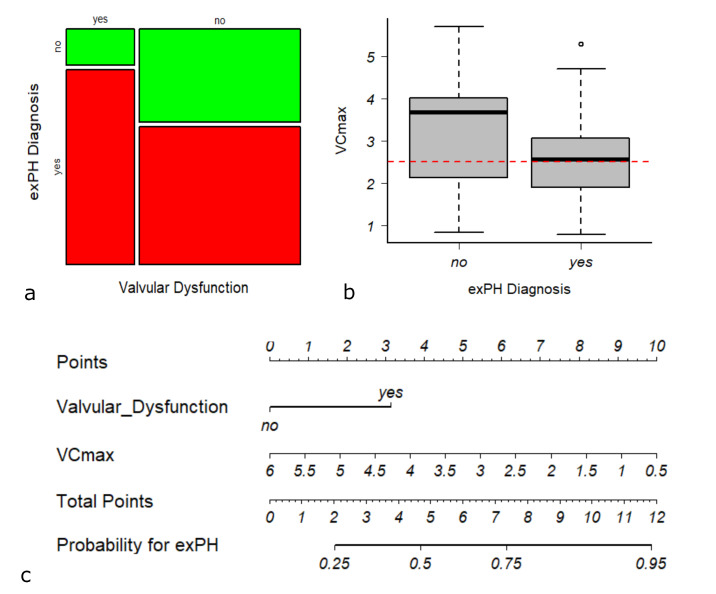
Vital capacity and valvular dysfunction in exercise pulmonary hypertension

The currently recommended cut-off for exPH in terms of mPAP is 30 mm Hg (82.8% of our patients demonstrated an exmPAP>30 mm Hg). Since we found a rather large difference between resting and exmPAP (Table 1) we increased the threshold to 35 mm Hg . A critical maximal performance level seems to be 100W, because at this workload we found a deflection point in exmPAP and exPAWP, as well as a continued decline of exPVR (Figure 1). 100W has also been suggested as a threshold to determine exaggerated (peripheral) blood pressure response during regular bicycle ergometry.^24^ Furthermore, peak performance of 100W was deemed to represent sufficient cardiorespiratory fitness for our elderly population. With higher workload, the probability of exPH may further decrease, represented by decreasing exmPAP, exPAWP and exPVR. Mean exPAWP at 100W was 18.3 mm Hg, which made us set the threshold to 18 mm Hg . At 100W PVR fell to 3WU, the current threshold to define pre-capillary PH with an mPAP>20 mm Hg at rest. Accounting for these workload haemodynamics and our multimorbid population, we chose the threshold of PVR>3WU as a cut-off, which is higher than the suggested PVR>2.10WU in patients>50 years in upright (as opposed to our supine) ergometry.^35^

According to our data and reflections we suggest a new score to define exPH incorporating the following four variables: (1) ExPAWP ≥18 mm Hg , (2) exmPAP≥35 mm Hg , (3) exPVR>3WU and (4) a maximal performance of ≤100Watt. For each criterion one point was awarded; exPH was diagnosed if three or four points were present. 7.4% received no points, 12.7% one, 16.8% two, 30.7% three, and 32.4% four points. Thus, 63.1% of patients were diagnosed with exPH.

### Step 3. Assessment of non-invasive variables to predict exPH

As standardised echocardiography, 6 MWD, and LFT with calculation of all variables was not available in all 244 exRHC patients within 24 hours of RHC investigation, the absolute numbers used for further analysis differed across the variables (Tables 3 and 4). Statistical bivariate analysis (Tables 3 and 4) with all available data sets was performed to elucidate the most useful variables for a non-invasive prediction model for exPH.

**Table 3:** Bivariate analysis of categorical non-invasive variables in patients with and without exercise pulmonary hypertension

		**exPH +**	**exPH -**	***P*** **value**
LVEF	>50%	74.5% (n=115)	67.8% (n=61)	*P* = .348
	40-50%	8.0% (n=12)	25.5% (n=23)	
	<40%	17.5% (n=27)	6.7% (n=6)	
Valvular dysfunction		yes:40.3% (n=62) no: 59.7% (n=92)	yes: 21.1% (n=19) no: 78.9% (n=71)	*P* = .004*
LTOT		yes: 15.6% (n=24) no: 84.4% (n=130)	yes: 7.8% (n=7) no: 92.2% (n=83)	*P* = .111
Sex		male: 47.4% (n=73) female: 52.6% (n=81)	male: 61.1% (n=55) female: 38.9% (n=35)	*P* = .046*
Hypertension		yes: 81.2% (n=125) no: 18.8% (n=29)	yes: 65.6% (n=59) no: 34.4% (n=31)	*P* = .009*
Smoking		yes: 23.4% (n=36) no: 76.6% (n=118)	yes: 18.9% (n=17) no: 81.1% (n=73)	*P* = .520
Afib		yes: 33.1% (n=51) no: 66.9% (n=103)	yes: 15.6% (n=14) no: 84.4% (n=76)	*P* = .003*
Diuretics		yes: 79.2% (n=122) no: 20.8% (n=32)	yes: 47.8% (n=43) no: 52.2% (n=47)	*P* < .001*
Diabetes		yes: 19.5% (n=30) no: 80.5% (n=124)	yes: 12.2% (n=11) no: 87.8% (n=79)	*P* = .159

Abbreviations: exPH, Exercise pulmonary hypertension; LVEF,Left ventricular ejection fraction ;LTOT, long-term oxygen treatment *Statistically significant

**Table 4:** Bivariate analysis of continuous non-invasive variables in patients with and without exercise pulmonary hypertension

		**n**	**Mean**	**SD**	**Median**	**IQR**	***P*** ** value**
Age, years	exPH +	154	69.9	11.0	71.0	12.8	*P* < .001*
	exPH -	90	62.2	11.7	62.0	16.0	
BMI, kg/m^2^	exPH +	154	28.6	4.4	28.5	5.2	*P* = .591
	exPH -	90	28.4	4.9	28.0	6.3	
sPAP, mm Hg	exPH +	84	44.9	14.0	44.0	18.3	*P* = .002*
	exPH -	27	34.3	14.9	30.0	19.0	
TAPSE, mm	exPH +	136	20.8	5.4	21.0	5.0	*P* = .078
	exPH -	78	22.2	3.5	22.0	2.8	
VC_max_, L	exPH +	99	2.6	0.8	2.6	1.1	*P* < .001*
	exPH -	49	3.3	1.1	3.6	1.7	
FEV1, L	exPH +	99	1.9	0.7	1.9	0.9	*P* = .002*
	exPH -	50	2.4	1.0	2.6	1.6	
DLCO (%)	exPH +	84	76.1	26.6	77.5	33.5	*P* = .020*
	exPH -	40	86.1	17.9	88.0	21.0	
Oxygen content, mL/dL	exPH +	144	12.8	1.5	12.8	1.8	*P* = .023*
	exPH -	86	13.2	1.3	13.2	1.5	
paO_2_, mm Hg	exPH +	148	66.0	10.6	67.0	15.3	*P* = .005*
	exPH -	88	70.6	9.4	70.5	12.0	
paCO_2,_ mm Hg	exPH +	148	37.5	5.4	37.0	6.0	*P* = .741
	exPH -	88	37.0	4.6	37.0	5.3	

Abbreviations:BMI, Body mass index; sPAP, systolic pulmonary artery pressure on echocardiography; TAPSE, tricuspid annular plane systolic excursion; DLCO, diffusion capacity of carbon monoxide *Statistically significant.

42.7% of our patients suffered from pulmonary disease, 100% from heart disease according to our definitions. The presence of pulmonary disease (*P* = .273) or coronary artery disease (*P* = .776) did not differentiate patients with or without exPH. Tables 3 and 4 provide data on baseline characteristics of patients with and without exPH as well as bivariate analysis of categorical (3) and continuous (4) variables.

Significant valvular dysfunction was mainly driven by tricuspid regurgitation (54.1%) and aortic stenosis (24.6%), followed by mitral (18.0%) and aortic regurgitation (3.3%). Maximal power differed significantly (*P* < .001) between males (92.3W±50.4, *P* < .001) and females (71.7W±37.2).

### Step 4. Calculation of a non-invasive prediction model for exPH

sPAP proved to be dominant in a logistic regression model suppressing the other variables. The Odds Ratio (OR) for the presence of exPH and an increase of 20 mm Hg in sPAP was 3.1 and the AUC was 0.70 (sensitivity 97.6% and specificity 22.2%, R^2^ 14.5%).

The problem of multicollinearity demonstrates that simultaneous integration of certain variables from bivariate analysis is not valid: sPAP showed significant associations with valvular dysfunction (*P* = .009) and age (*P* = .016; r=0.229), while age itself was significantly associated with valvular dysfunction (*P* < .001). Thus, an alternative model without sPAP was calculated based on the significant variables from bivariate analysis (atrial fibrillation was left out due to its known interdependence with hypertension) and stepwise forward/backward selection modelling was performed, leaving only VC_max_, age, and valvular dysfunction (AUC 0.75, R^2^ 26.1%). This model increased specificity to 44.2% with little loss of sensitivity (94.3%).

We reduced our model to VC_max_ and valvular dysfunction (Table 5) because of a clinically established association between age and VC_max_. Information on VC_max_ within 24 hours of RHC was available in 131 patients (53.7% of 244, baseline information on the 131 patients included in the final model are shown in Supplement 1). Bivariate analysis of variables in the 131 patients did not change the selection of factors for the prediction model (Supplement 1, Table 4) and a sensitivity of 93.2% (CI: 85.9-96.8%) and a specificity of 44.2% (30.4-58.9%; AUC 0.73, R^2^ 22.1%) were yielded. The OR was 3.6 (CI: 1.4-10.8) for valvular dysfunction and 2.1 (CI: 1.4-3.3) for 1l loss in VC_max_ (Table 5).

**Table 5:** Logistic model for estimation of exercise pulmonary hypertension

	**Coefficient**	**Standard error**	***P*** ** value**
Intercept	3.855	0.794	<.001*
VC_max_	-0.750	0.212	<.001*
Valvular dysfunction	-1.290	0.514	0.012*

*Statistically significant

The analysis of the individual variables showed that valvular dysfunction was a specific parameter (specificity 86.0% and sensitivity 37.5%), while VC_max_ was more sensitive. At a cut-off of 2.52 l VC_max_ a sensitivity of 53.4% and a specificity of 39.5% were obtained.

Sub-analysis of the group without resting PH (n=80) demonstrated a sensitivity of 90.5% (CI: 77.9-96.2%) and a specificity of 50.0% (CI: 34.8-65.2%), while a calculation in the group with resting PH (n=51) showed a sensitivity of 95.7% (CI: 85.5-98.8%); specificity could not be reasonably calculated due to the low case number.

## Discussion

In our alternative model a lower number of patients qualified for exPH than in the current suggestion of TPR>3 mm Hg/L/min and mPAP>30 mm Hg (63.1% vs. 78.3%), which is likely to reduce the number of false positives in our elderly population (mean age 67.0 years±11.9). Patients with exPH were significantly older, had lower VC_max_, higher sPAP, more valvular dysfunctions, diuretics, atrial fibrillation, and hypertension. We suggest our new scoring system for defining exPH in elderly patients with low output during exercise (CO<10 L/min).

Our study investigated a significantly different population of patients compared to a French^19^ (mean age in the group with left heart disease was 61.1 years, lung disease 55.8 years and healthy controls 46.1 years; a total of 169 patients) and American^22^ (mean age 57 years and preserved ejection fraction, n=714) study. In contrast, our population consisted of more elderly (69.9 years±11.0 in exPH patients and 62.2 years±11.7 in those without exPH) multi-morbid patients (100% suffering from heart and 42.7% from pulmonary disease). We did not find evidence that resting left or right ventricular function had an influence on the presence of exPH. More subtle methods to detect differences, such as strain analysis, may be needed. Furthermore, a higher number (40.3% vs. 20.0%) of our exPH patients had significant valvular disease (especially functional tricuspid regurgitation) compared to a previous study.^19^

Additionally, we used a different algorithm for study inclusion than previous works: All patients who received exRHC were included in the analysis to define alternative thresholds independent of the presence of resting PH (most of our patients had borderline PH at rest). In clinical practice we perform exRHC in patients with moderate PH at rest to analyse the hemodynamic response to low-intensity exercise. This is highly relevant to understand patients’ workload and the stress on pulmonary vasculature during daily (low-intensity) activities, such as walking, shopping, or doing the household. This approach can be seen analogous to established exercise testing with bicycle ergometry, which is also performed in patients with overt arterial hypertension at rest to analyse (peripheral) blood pressure response during exercise. Due to the low exercise time of our exRHC exams it is unlikely that patients will suffer harm, even in the case of (moderate) resting PH (no adverse events were observed during exPH). However, as the increase of PH (and thus hypoxemia) during exercise may amplify anaerobic peripheral muscle work and lactate accumulation, further studies might integrate exRHC with capillary (or even muscle) lactate measurements to better understand the metabolic, on top of the cardio-circulatory, burden. Different testing protocols (ramp vs. continuous testing) might be needed in a multimorbid population.^36^

It has been postulated that the suggested algorithm of exmPAP>30 mm Hg and exTPR>3 mm Hg/L/min (may be seen as current gold standard) might be problematic in elderly patients.^23^ Muscular limitations leading to exCO<10 L/min may lead to high exTPR-values but lower exmPAP. This was exactly the case in our population, with 90.7% showing an exTPR >3 mm Hg/L/min and a mean exCO of 7.3 L/min. Thus, exTPR may increase the number of false positives in the elderly who fail to generate an exCO>10 L/min. Especially in our elderly population with lower exCO, we believe exTPR to be a poor discriminator for exPH and therefore excluded it in our model. We found the deflection points of exmPAP and exPAWP at a performance of 100W, which supports the decision to take a cut-off of 100W workload for the diagnostic score. The drop of exmPAP and PAWP with higher performance may reflect better vascular compliance in patients with higher cardiorespiratory fitness (CRF, expressed by peak power).^37-39^ A PVR threshold >3WU has been suggested at rest^17^ and exercise^19^ and was reached at 100W (3.8WU±1.5). Across all variables CRF turned out to play a major role in the definition of exPH. Similarly to CPET, CRF should also be implemented in scores used to define exPH, because higher CRF is associated with the improvement of haemodynamics in PH.^40,41^ We did not find body mass index (BMI) to have a significant impact on the presence of exPH, which has already been shown in the literature^19^ This does not contradict the claim for the integration of CRF into exPH definitions, since BMI only reflects a body mass-to-body surface ratio and does not consider actual muscle mass or body composition.

We aimed to suggest a clinically reasonable cut-off by using deflection points (as a marker for better pulmonary compliance with higher peak workload) as thresholds (Figure 1): The workload at a relevant decrease of PAP and PVR (due to vascular compliance after an initial increase) was considered reasonable as a cut-off. The threshold for the step-wise decline of PVR [<3WU recommended as a threshold in current guidelines on resting PH] was adapted from current recommendations.^17,22^

We believe that it is reasonable that the workload achieved should, or even must, be included in guidelines on exPH. In conventional bicycle ergometry (peripheral as opposed to pulmonary), exercise hypertension is often defined as a systolic blood pressure (SBP)>200 mm Hg at a workload of 100W and has been associated with increased cardiovascular mortality. This is a practical approach, although data is lacking on this account. ^42^ However, from a pathophysiological and clinical perspective there is a great difference in an SBP of 200 mm Hg at 100W (clearly a problem which should lead to an increase in blood pressure medication, both in the absence or presence of resting hypertension), or 200 mm Hg at a workload of 300W in a trained athlete at peak performance with adequate (pulmonary and peripheral) vascular compliance and immediate decline of pressure after exercise termination. In our point of view the same mechanism should be applied to the definition of exPH, independent of resting PH. Our study provides a first impulse. Clearly, there need to be further studies to refine this.

Since RHC in elderly patients may not always be feasible, we calculated a prediction model of exPH with non-invasive variables from echocardiography and lung function testing. sPAP was the most sensitive marker for exPH (sensitivity 97.6%), but at the price of very low specificity (22.2%). The OR for an increase of 20 mm Hg sPAP was 3.1. By considering the multicollinearity of variables (especially valvular dysfunction and sPAP), we used a combinatorial model with VC_max_ and valvular dysfunction to predict exPH (sensitivity 93.2%, specificity 44.2%; AUC 0.73), with associated OR of 2.1 for a 1l loss in VC_max_ and 3.6 for valvular dysfunction. A sub-analysis of the group with and without resting PH did not yield a clinically relevant difference in test strength. Valvular dysfunction by itself (mainly driven by tricuspid regurgitation and aortic stenosis) proved to be very specific (86.0%, see Figure 2a). Despite its potential to discriminate patients with exPH in the combinatorial model, definition of a suitable cut-off for VC_max_ alone is difficult and was set at 2.52 l (Figure 2b). A nomogram was drawn to visualise a prognostic score for estimating the probability of exPH (Figure 2c). From a clinical perspective, a high sensitivity ensures that the majority of true positives are detected. Therefore, application of the combinatorial model in conjunction with high sPAP-values provides a quite secure basis for accurate non-invasive detection of exPH. On the other hand, exclusion of relevant valvular dysfunction in transthoracic echocardiography makes exPH rather unlikely: A patient without valvular dysfunction (0 points) and a VC_max_ of 3.5l (4.5 points) would have less than 50% probability of exPH (4.5 points in total). On the other hand if the same patient suffered from valvular disease, probability would rise to 75% (three points for valvular dysfunction). Non-invasive evaluation of lung function and echocardiography for exPH estimation is appealing, since data can easily be acquired.

We are aware that the test strength is not sufficient to suggest our prediction model as a gold standard to replace invasive diagnostics. However, its high sensitivity may prove useful as a screening test to exclude exPH,^43^ but has to be verified in different patient populations with mild and severe disease. In the future, the relatively moderate specificity of our prediction model could be improved by changing the cut-off values to higher levels, for instance to an exmPAP of 40 mm Hg and an exPAWP of 20 mm Hg together with a higher peak performance (e.g., 125W). Further data is needed on this as well as on the issue of treatment initiation in isolated exPH. ^44^

ExRHC is a challenging procedure and needs training and routine to acquire solid data. Technical obstacles of RHC have to be discussed as well. For instance, it may be difficult to immediately reach the wedge position at peak performance and high pulmonary artery pressures, leading to dislodgement of the catheter to more proximal areas. Failure of immediate ‘wedging’ may lead to false low values. The inter-study comparison of data may also be hampered by the sequence of data collection: we have established the following sequence of data acquisition at peak performance: (1) PA-values, (2) PAWP, and (3) PA position to obtain CO. This procedure may be done differently in other institutions with a certain degree of variation in values.

We are aware of some limitations of this study, which mainly concern the rather low number of data sets for the prediction model (n=131), although seminal papers on exRHC have similar case counts and study design.^19^ Similar to existing literature, our heart failure group mainly consisted of patients with preserved ejection fraction.^19^ We have little data on patients with highly reduced ejection fraction. Categorisation of our heart failure group (as well as the group with pulmonary disease) was arbitrary and may include entities with different pathophysiologies. Additionally, (stress) echocardiography and gas exchange were not applicable during RHC, this could have provided more information on the etiology of disease. Our cut-off values for the score were oriented on the dynamics across performance (using the pathophysiologically-oriented deflection point method instead of a statistically-oriented ROC-analysis) and may not apply to a younger population. Due to the retrospective nature of our study, our data is subject to bias and needs to be validated in a prospective cohort of elderly patients. Unfortunately, we do not have a follow-up of our patients over time to see whether our model translates into higher mortality rates in exPH patients.

## Conclusion

We provide an alternative score than the currently proposed definition of exPH (exTPR>3 mm Hg/L/min +exmPAP>30 mm Hg) for elderly patients with concomitant cardiopulmonary diseases; this reduces the number of exPH diagnoses in this population. Our score integrates peak performance as an essential variable of exercise testing. Only a combinatorial approach, such as ours, can pay tribute to changing vascular compliance during exercise to differentiate physiological alterations from actual disease. Additionally, we identify VC_max_ and valvular dysfunction as significant factors for a non-invasive prediction model to estimate and screen for exPH in elderly patients. This may allow clinicians to circumvent invasive procedures in this high-risk group and will help to stratify patients who may qualify for early treatment initiation in isolated exPH.

## Acknowledgments

We thank Mr Kelly for native proofreading of the manuscript. We are also grateful to Mr Marcus Möllenberg, who conducted a number of right heart catheterisations and echocardiographies. Special thanks go to Prof. Tienush Rassaf for proofreading the manuscript.

## Competing interest

The authors declare that they have no competing interests. The results have not been presented elsewhere.

## Ethics approval

Patient or their legal guardians signed consent for the right heart catheter procedures. This study was conducted in accordance with the amended Declaration of Helsinki, and ethical approval was obtained (Ethics Committee University Munster, Germany, 2020-417-f-S).

## Funding

No funding was obtained for this study.

## References

[1] Hoeper MM, Kramer T, Pan Z, Eichstaedt CA, Spiesshoefer J, Benjamin N (2017). Mortality in pulmonary arterial hypertension: prediction by the 2015 European pulmonary hypertension guidelines risk stratification model. Eur Respir J.

[2] Lancellotti P, Magne J, Donal E, O’Connor K, Dulgheru R, Rosca M (2012). Determinants and prognostic significance of exercise pulmonary hypertension in asymptomatic severe aortic stenosis. Circulation.

[3] Lancellotti P, Magne J, Dulgheru R, Ancion A, Martinez C, Piérard LA (2015). Clinical significance of exercise pulmonary hypertension in secondary mitral regurgitation. Am J Cardiol.

[4] Magne J, Lancellotti P, Piérard LA (2010). Exercise pulmonary hypertension in asymptomatic degenerative mitral regurgitation. Circulation.

[5] Magne J, Lancellotti P, Piérard LA (2014). Exercise testing in asymptomatic severe aortic stenosis. JACC Cardiovasc Imaging.

[6] Kanwar MK, Gomberg-Maitland M, Hoeper M, Pausch C, Pittrow D, Strange G (2020). Risk stratification in pulmonary arterial hypertension using Bayesian analysis. Eur Respir J.

[7] Oliveira RKF, Faria-Urbina M, Maron BA, Santos M, Waxman AB, Systrom DM (2017). Functional impact of exercise pulmonary hypertension in patients with borderline resting pulmonary arterial pressure. Pulm Circ.

[8] Faria-Urbina M, Oliveira RKF, Segrera SA, Lawler L, Waxman AB, Systrom DM (2018). Impaired systemic oxygen extraction in treated exercise pulmonary hypertension: a new engine in an old car?. Pulm Circ.

[9] Medarov BI, Jogani S, Sun J, Judson MA (2017). Readdressing the entity of exercise pulmonary arterial hypertension. Respir Med.

[10] Segrera SA, Lawler L, Opotowsky AR, Systrom D, Waxman AB (2017). Open label study of ambrisentan in patients with exercise pulmonary hypertension. Pulm Circ.

[11] Wallace WD, Nouraie M, Chan SY, Risbano MG (2018). Treatment of exercise pulmonary hypertension improves pulmonary vascular distensibility. Pulm Circ.

[12] Kubo K, Ge RL, Koizumi T, Fujimoto K, Yamanda T, Haniuda M (2000). Pulmonary artery remodeling modifies pulmonary hypertension during exercise in severe emphysema. Respir Physiol.

[13] Sanders JL, Han Y, Urbina MF, Systrom DM, Waxman AB (2019). Metabolomics of exercise pulmonary hypertension are intermediate between controls and patients with pulmonary arterial hypertension. Pulm Circ.

[14] Oliveira RK, Waxman AB, Agarwal M, Badr Eslam R, Systrom DM (2016). Pulmonary haemodynamics during recovery from maximum incremental cycling exercise. Eur Respir J.

[15] Zou RH, Wallace WD, Nouraie SM, Chan SY, Risbano MG (2020). Lower DLco% identifies exercise pulmonary hypertension in patients with parenchymal lung disease referred for dyspnea. Pulm Circ.

[16] Singh I, Rahaghi FN, Naeije R, Oliveira RKF, Vanderpool RR, Waxman AB (2019). Dynamic right ventricular-pulmonary arterial uncoupling during maximum incremental exercise in exercise pulmonary hypertension and pulmonary arterial hypertension. Pulm Circ.

[17] Galiè N, Hoeper MM, Humbert M, Torbicki A, Vachiery JL, Barbera JA (2009). Guidelines for the diagnosis and treatment of pulmonary hypertension: the Task Force for the Diagnosis and Treatment of Pulmonary Hypertension of the European Society of Cardiology (ESC) and the European Respiratory Society (ERS), endorsed by the International Society of Heart and Lung Transplantation (ISHLT). Eur Heart J.

[18] Hatano S, Strasser T. Primary Pulmonary Hypertension: Report on a WHO Meeting. Geneva: WHO; 1973.

[19] Herve P, Lau EM, Sitbon O, Savale L, Montani D, Godinas L (2015). Criteria for diagnosis of exercise pulmonary hypertension. Eur Respir J.

[20] Kovacs G, Berghold A, Scheidl S, Olschewski H (2009). Pulmonary arterial pressure during rest and exercise in healthy subjects: a systematic review. Eur Respir J.

[21] Kovacs G, Herve P, Barbera JA, Chaouat A, Chemla D, Condliffe R (2017). An official European Respiratory Society statement: pulmonary haemodynamics during exercise. Eur Respir J.

[22] Ho JE, Zern EK, Lau ES, Wooster L, Bailey CS, Cunningham T (2020). Exercise pulmonary hypertension predicts clinical outcomes in patients with dyspnea on effort. J Am Coll Cardiol.

[23] Kovacs G, Avian A, Olschewski H (2016). Proposed new definition of exercise pulmonary hypertension decreases false-positive cases. Eur Respir J.

[24] Mariampillai JE, Liestøl K, Kjeldsen SE, Prestgaard EE, Engeseth K, Bodegard J (2020). Exercise systolic blood pressure at moderate workload is linearly associated with coronary disease risk in healthy men. Hypertension.

[25] Simonneau G, Montani D, Celermajer DS, Denton CP, Gatzoulis MA, Krowka M (2019). Haemodynamic definitions and updated clinical classification of pulmonary hypertension. Eur Respir J.

[26] Criée CP, Baur X, Berdel D, Bösch D, Gappa M, Haidl P (2015). [Standardization of spirometry: 2015 update Published by German Atemwegsliga, German Respiratory Society and German Society of Occupational and Environmental Medicine]. Pneumologie.

[27] Schneider M, Aschauer S, Mascherbauer J, Ran H, Binder C, Lang I (2019). Echocardiographic assessment of right ventricular function: current clinical practice. Int J Cardiovasc Imaging.

[28] Lang RM, Badano LP, Mor-Avi V, Afilalo J, Armstrong A, Ernande L (2015). Recommendations for cardiac chamber quantification by echocardiography in adults: an update from the American Society of Echocardiography and the European Association of Cardiovascular Imaging. J Am Soc Echocardiogr.

[29] Baumgartner H, Hung J, Bermejo J, Chambers JB, Edvardsen T, Goldstein S (2017). Recommendations on the echocardiographic assessment of aortic valve stenosis: a focused update from the European Association of Cardiovascular Imaging and the American Society of Echocardiography. J Am Soc Echocardiogr.

[30] Zoghbi WA, Adams D, Bonow RO, Enriquez-Sarano M, Foster E, Grayburn PA (2017). Recommendations for noninvasive evaluation of native valvular regurgitation: a report from the American Society of Echocardiography developed in collaboration with the Society for Cardiovascular Magnetic Resonance. J Am Soc Echocardiogr.

[31] Opotowsky AR, Hess E, Maron BA, Brittain EL, Barón AE, Maddox TM (2017). Thermodilution vs estimated Fick cardiac output measurement in clinical practice: an analysis of mortality from the Veterans Affairs Clinical Assessment, Reporting, and Tracking (VA CART) program and Vanderbilt University. JAMA Cardiol.

[32] Cigarroa RG, Lange RA, Williams RH, Bedotto JB, Hillis LD (1989). Underestimation of cardiac output by thermodilution in patients with tricuspid regurgitation. Am J Med.

[33] R Core Team. R: A language and environment for statistical computing. R Foundation for Statistical Computing. 2020 [12 May 2020]; Available from: https://www.r-project.org/.

[34] Jr FEH. rms: Regression Modeling Strategies. R package version 5.1-4. 2019 [12 May 2020]; Available from: https://cran.r-project.org/web/packages/rms/index.html.

[35] Oliveira RK, Agarwal M, Tracy JA, Karin AL, Opotowsky AR, Waxman AB (2016). Age-related upper limits of normal for maximum upright exercise pulmonary haemodynamics. Eur Respir J.

[36] Tschakert G, Hofmann P (2013). High-intensity intermittent exercise: methodological and physiological aspects. Int J Sports Physiol Perform.

[37] Albin EE, Brellenthin AG, Lang JA, Meyer JD, Lee DC (2020). Cardiorespiratory fitness and muscular strength on arterial stiffness in older adults. Med Sci Sports Exerc.

[38] Deiseroth A, Streese L, Köchli S, Wüst RS, Infanger D, Schmidt-Trucksäss A (2019). Exercise and arterial stiffness in the elderly: a combined cross-sectional and randomized controlled trial (examin age). Front Physiol.

[39] Perissiou M, Bailey TG, Windsor M, Nam MCY, Greaves K, Leicht AS (2018). Effects of exercise intensity and cardiorespiratory fitness on the acute response of arterial stiffness to exercise in older adults. Eur J Appl Physiol.

[40] Ehlken N, Lichtblau M, Klose H, Weidenhammer J, Fischer C, Nechwatal R (2016). Exercise training improves peak oxygen consumption and haemodynamics in patients with severe pulmonary arterial hypertension and inoperable chronic thrombo-embolic pulmonary hypertension: a prospective, randomized, controlled trial. Eur Heart J.

[41] Richter MJ, Grimminger J, Krüger B, Ghofrani HA, Mooren FC, Gall H (2017). Effects of exercise training on pulmonary hemodynamics, functional capacity and inflammation in pulmonary hypertension. Pulm Circ.

[42] Schultz MG, La Gerche A, Sharman JE (2017). Blood pressure response to exercise and cardiovascular disease. Curr Hypertens Rep.

[43] Eusebi P (2013). Diagnostic accuracy measures. Cerebrovasc Dis.

[44] Hoeper MM (2020). Exercise pulmonary hypertension is back. J Am Coll Cardiol.

